# Analysis of trichoscopic images using deep neural networks for the diagnosis and activity assessment of alopecia areata – a retrospective study

**DOI:** 10.1111/ddg.15847

**Published:** 2025-09-30

**Authors:** Raffaele Dante Caposiena Caro, Victoria Orlova, Nicola Di Meo, Iris Zalaudek

**Affiliations:** ^1^ Dermatology Clinic Hospital Maggiore of Trieste University of Trieste Trieste Italy

**Keywords:** Alopecia areata, Artificial Intelligence, Deep Learning, Dermatology, Machine Learning, Trichology, Trichoscopy

## Abstract

**Background and objectives:**

Alopecia areata (AA) is an autoimmune disease that provokes hair loss. The diagnosis is made clinically with the support of trichoscopy. However, trichoscopy requires specialized training. Deep learning models may support the diagnosis and management of AA. The aim of this study is to develop a deep learning framework to diagnose AA and to determine the AA level of activity.

**Patients and methods:**

A retrospective analysis of trichoscopic images collected from patients with scalp diseases and healthy controls was conducted to develop a two‐step deep learning framework. In Step‐1, the model aimed to distinguish AA disease from both other scalp diseases and control healthy subjects. In Step‐2, we intended to train a model that recognized the AA level of activity dividing the AA dataset into active, inactive, and regrowth.

**Results:**

In Step‐1 an overall accuracy of 88.92% and an F1 score of 88.17% were achieved with an AA discriminatory capacity of 90.98%. In Step‐2 an accuracy of 83.33% and an F1 score of 83.36% were reached.

**Conclusions:**

Our study highlighted for the first time the potential use of artificial intelligence in the diagnosis and staging of AA allowing more accurate diagnoses and better care.

## INTRODUCTION

Alopecia areata (AA) is an autoimmune, non‐scarring hair‐loss disease characterized by sudden, patchy, and usually asymptomatic hair‐loss. It primarily affects hair follicles on the scalp but can also involve body hair.^1^, ^2^ AA affects approximately 2% of the general population.^1^, ^2^ However, several subtypes of AA have been described, including patchy alopecia, alopecia totalis (AT), alopecia universalis (AU), ophiasis, sisaipho, diffuse alopecia areata (DAA), alopecia areata incognita (AAI), and Marie Antoinette and Thomas More syndrome.^1^, ^2^ AA exhibits variable progression. Initial bald patches may expand, multiply, or resolve spontaneously with hair regrowth within a few months. Spontaneous recovery occurs in 34–50% of cases within one year, though many patients experience relapses. The relapse rate ranges from 30% to 52%, with most relapses (79%) occurring within the first 4 years.^1^, ^2^ About 14–25% of patients progress to AT or AU, and full recovery is rare (< 10%).^1^, ^2^

Nail changes, particularly in more severe forms such as AU and AT, are common in AA. These changes are often subtle and asymptomatic, with pitting and trachyonychia being the most frequent yet often overlooked. The prevalence of nail abnormalities in patients with AA is likely underestimated, with reported rates ranging from 7 % to 66 % and an estimated mean of about 30 %.^3^ Another significant aspect of AA is psychological impact, leading to an increased risk of stress, anxiety, and depression. Notably, AA has been found to raise the likelihood of developing major depressive disorder by 34%. Additionally, suicide attempts are the highest among patients with AU and DAA.^4^ For instance, one study reported suicidal ideation in 60% of AU patients, more than three times higher than in patients with localized alopecia (18%).^4^, ^5^

The diagnosis of AA is primarily clinical. Its differential diagnoses include both non‐scarring and scarring alopecias as well as genetic conditions associated with hair‐loss.^1^, ^2^ Patchy AA should be distinguished from several conditions including tinea capitis, trichotillomania, temporal triangular alopecia, and scarring alopecia such as lichen planopilaris (LPP). In cases of frontal involvement or ophiasis, frontal fibrosing alopecia (FFA) – a subtype of LPP – must be carefully considered. FFA presents as a band‐like frontotemporal alopecia with eyebrow loss and, in rare cases, occipital involvement. Moreover, DAA and AAI can be misdiagnosed as telogen effluvium (TE) or female pattern hair‐loss (FPHL). The speed of hair‐loss is also a key diagnostic factor. Sudden onset suggests TE, AA, or anagen effluvium (e.g., chemotherapy‐induced), whereas gradual onset is more typical of androgenetic alopecia (AGA) or scarring alopecias (e.g., LPP).^1^, ^2^ Due to numerous differential diagnoses, histological examination remains the gold standard in unclear cases, allowing differentiation between AA and other alopecias.^1^, ^2^ Also trichoscopy, a non‐invasive dermoscopy technique for scalp and hair analysis, plays a crucial role in diagnosing AA.^6^ It aids in evaluating inflammatory activity, severity markers, treatment response, and prognostic factors, making it an essential tool in trichology.^7^ However, trichoscopy requires specialized training.^8^ Developing computer‐aided diagnostic tools could assist dermatologists in diagnosing AA, differentiating it from other alopecias, and identifying trichoscopic markers of hair regrowth and disease activity to optimize treatment.^9^ Mild AA, with *Severity of Alopecia Tool* (SALT) ≤ 20, is commonly treated with corticosteroids (topical or intralesional) and rarely with contact immunotherapy, (e.g., squaric acid dibutyl ester). In moderate to severe AA (SALT > 20), systemic therapies such as oral corticosteroids or Janus kinase (JAK) inhibitors are indicated.^10^, ^11^

Machine learning (ML) and deep learning (DL), key components of artificial intelligence (AI), are increasingly being applied in dermatology.^5^ AI supports diagnosis prediction and treatment outcome assessment.^9^ Recently, AI‐based models for AA diagnosis and severity assessment have gained attention. Most studies focused on clinical scalp and hair images classification, demonstrating promising results in distinguishing AA from healthy conditions and assessing severity. However, only a few studies explored AI applications using trichoscopic images. Some developed automated SALT index calculation frameworks using segmentation techniques. Others focused on detecting hair density, diameter, and additional parameters relevant to the early diagnosis of AA and other hair disorders.^12^, ^13^, ^14^, ^15^, ^16^, ^17^, ^18^, ^19^, ^20^, ^21^, ^22^, ^23^, ^24^, ^25^, ^26^, ^27^, ^28^, ^29^, ^30^, ^31^, ^32^, ^33^

This study aimed to develop a DL framework using videodermoscopy images to diagnose AA and assess disease activity, enhancing clinical decision‐making for healthcare professionals with proven DL architectures for image classification.

## PATIENTS AND METHODS

### Subject enrollment

The study was conducted retrospectively on images captured during routine clinical care from all patients with scalp diseases treated at our institution between March 2022 and March 2024, along with a group of Healthy Volunteers (HV). HV were volunteer healthcare professionals from our department. Subjects meeting the criteria reported in Table 1 were included. Subjects were categorized into five groups: *(1)* patients with active AA, *(2)* patients with inactive or chronic AA, *(3)* patients with AA in regrowth phase, *(4)* patients with other common scalp diseases, including folliculitis decalvans (FD), FFA, LPP, and AGA, *(5)* HV. AA classification was based on trichoscopic findings, as described in the literature (Table 2).^34^, ^35^ Similarly, the diagnoses of other scalp diseases were made according to established literature.^36^, ^37^, ^38^, ^39^, ^40^, ^41^, ^42^, ^43^, ^44^

**TABLE 1:** Subject enrollment criteria.

Inclusion criteria	‐ Adults (> 18 years) with trichological disorders ‐ Availability of videodermoscopic images ‐ Signed informed consent from our hospital, allowing the use of clinical data for research purposes, including genetic, biometric, and photographic data for clinical and epidemiological research.
Exclusion criteria	‐ Subjects < 18 years ‐ Lack of informed consent (only for healthy volunteers)

**TABLE 2:** Dataset inclusion and exclusion criteria for images.

Active alopecia areata	‐ Yellow dots ‐ Exclamation‐mark hairs ‐ Broken hairs ‐ Tapered hairs ‐ Pohl‐Pinkus constrictions ‐ Coudability hairs
Inactive alopecia areata	‐ Yellow dots ‐ Vellus hair ‐ Empty follicular openings ‐ White dots
Regrowing alopecia areata	‐ Upright regrowing hairs ‐ Pigtail hairs ‐ Vellus hairs

### Dataset

In this study, we used a dataset of trichoscopic images obtained during physical examinations at our Ambulatory of Adnexal Diseases, all captured by the same investigator (RDC). Videodermoscopy images were acquired with the Medicam 1000® video‑dermoscope (FotoFinder Systems GmbH, Germany; version 3.1.3.0 [x64]) and subsequently employed to train and validate the algorithm. Digital trichoscopic images were collected from all affected scalp areas. Each image was reviewed and classified by an expert trichologist with 10 years of experience at Trieste University Hospital (RDC), who defined the classification criteria, together with two additional independent experts (IZ and NdM). Any disagreements were resolved through discussion among the experts. Inclusion criteria for the dataset images are reported in Table 3. The dataset contains images categorized into three levels of AA activity: *(1)* active, *(2)* inactive, and *(3)* regrowth. Additionally, the dataset included images of four other scalp diseases: FD, FFA, LPP, AGA, and HV.

**TABLE 3:** Alopecia areata classification according to trichoscopy findings.

Inclusion criteria	‐ Diagnosis confirmed by all three experts, ‐ Diagnosis confirmed by histology in unclear cases (i.e., when expert consensus was not reached), ‐ In‐focus images, ‐ Images collected only at 20‐fold magnification (equivalent to a field of view of 0.903 cm^2^). ‐ Signed informed consent for clinical research purposes.
Exclusion criteria	‐ Cases without agreement among all three experts and lacking histological confirmation, ‐ Out‐of‐focus images, ‐ Images taken at magnifications other than 20‐fold (e.g., 40‐fold), ‐ Duplicate images. ‐ Absence of signed informed consent.

We developed a two‐step DL framework. Step‐1 was trained to distinguish AA from other scalp diseases and HV. For this step, the dataset was divided into five groups of images: *(1)* AA, *(2)* FD, *(3)* FFA and LPP, *(4)* AGA, and *(5)* HV. In Step‐2, we developed a model to distinguish the different AA activity states. For this purpose, the dataset containing the three AA subgroups (active, inactive, and regrowth) was utilized.

### Data normalization and augmentation

**FIGURE 1:**
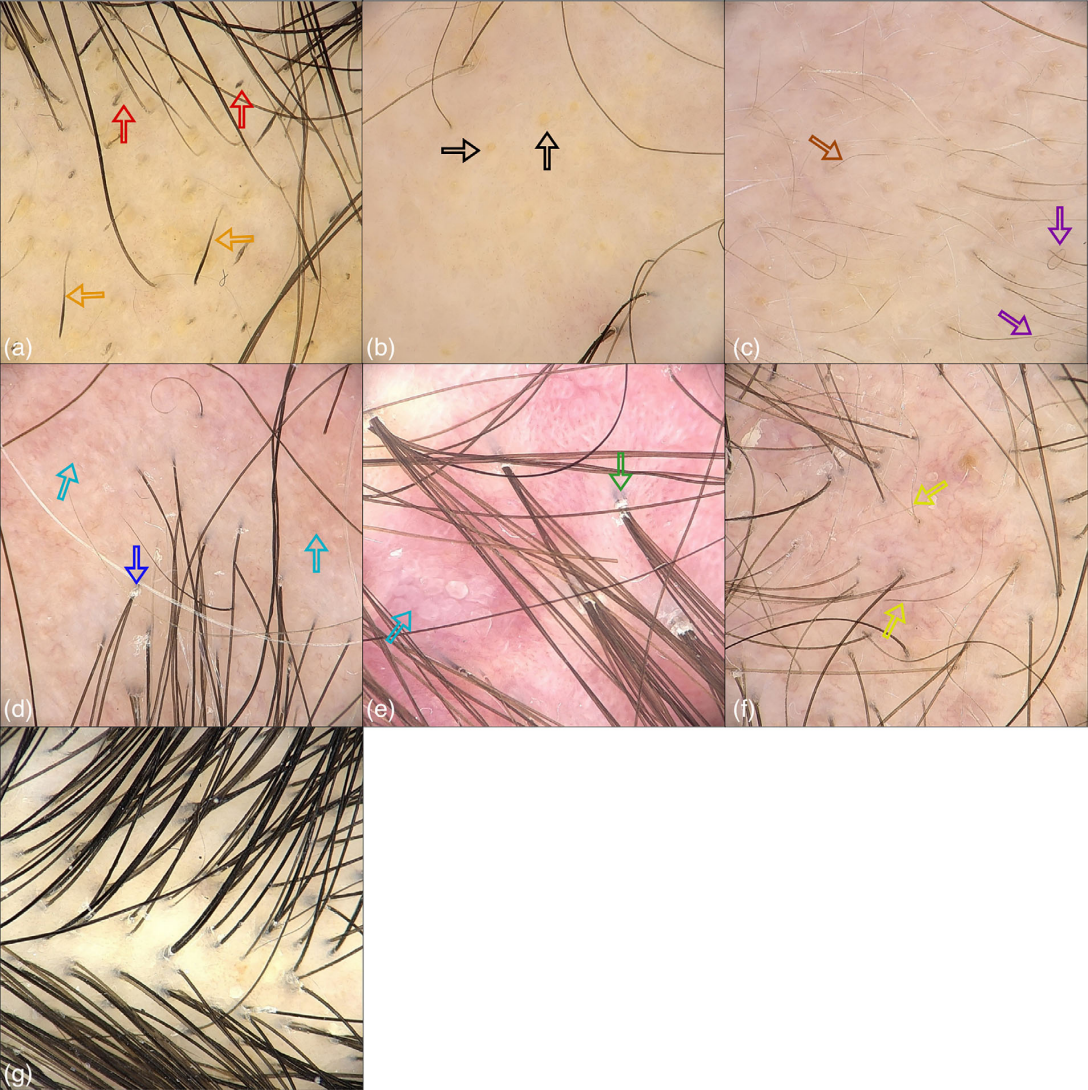
Example images from the different disease classes included in the algorithms. (a) Active alopecia areata, black dots (red arrows), exclamation mark hairs (orange arrows); (b) Chronic‐inactive alopecia areata, yellow dots (black arrow); (c) Regrowing alopecia areata, vellus hairs (brown arrows), circle hairs (purple arrows); (d) Lichen planopilaris, perifollicular scales (blue arrow), scarring areas (sky‐blue arrows); (e) Folliculitis decalvans, tufted hairs with perifollicular scales (green arrow), scarring areas (sky‐blue arrows); (f) Androgenetic alopecia, thinning hair (yellow arrow). In Step‐1, panels (a), (b), and (c) were aggregated in the class alopecia areata, (d) in the class fibrosing frontal alopecia and lichen planopilaris, (e) in the class folliculitis decalvans, (f) in the class androgenetic alopecia, and (g) in the class healthy controls. For Step‐2, the images in panels (a), (b), and (c) represent three separate classes.

**FIGURE 2:**
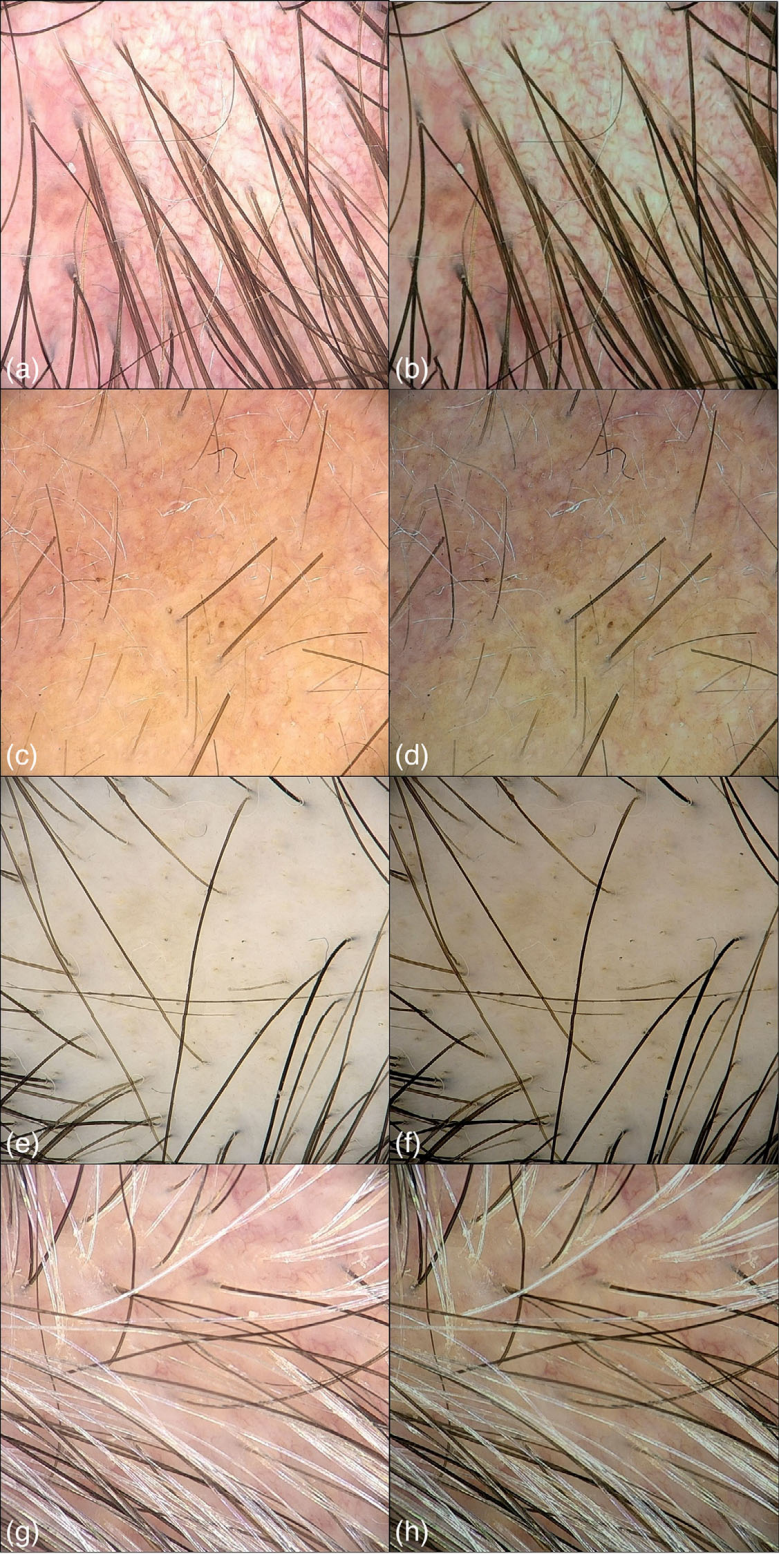
(a), (c), (e), (g) Scalp videodermatoscopy images before normalization; (b), (d), (f), (h) scalp videodermatoscopy images after normalization.

Only digital trichoscopic images of affected and healthy scalp areas that met all inclusion criteria, and no exclusion criteria were included (Figure 1). The images were cropped to remove camera shadows and eliminate noise, improving clarity and focus on the scalp areas. However, skin color may vary depending on lighting conditions and patient characteristics. To address this issue, normalization was applied to the data. The primary goal of normalization is to minimize brightness and skin tone variability, ensuring that the model focuses on relevant features rather than being influenced by variations in lighting and skin color (Figure 2).^25^, ^45^, ^46^ Details are reported in online supplementary Figures S1 and S2.

The two datasets were divided into a training set (80%) for model development and a test set (20%) for validation, ensuring that both subsets maintained the same class distribution as the original datasets. The main limitation of the dataset is the relatively small number of images, with some classes being underrepresented. In the first model, class imbalance is observed in FD and AGA clusters, while in the second model, the regrowing AA class is more represented than others. To balance the dataset and expand the overall number of images, data augmentation was applied to the training and test datasets. This approach involved various transformations of existing data, including random image rotation and color distortion obtained using the Principal Component Analysis (PCA) approach.^45^, ^46^, ^47^ Details are reported in Online Supplement.

### Model description

In our research, we utilized three architectures of convolutional neural network (CNN) models: Residual Neural Network (ResNet152), Dense Convolutional Network (DenseNet169), and EfficientNetB0.^48^, ^49^, ^50^, ^51^, ^52^ A description of the models and parameter details are reported in Online Supplement and online supplementary Table S2.

### Transfer learning

The limited size of the available image dataset makes it challenging to train a model from scratch, despite a substantial increase in the training data through augmentation. To overcome the issue of limited data, we employed the theory of transfer learning (TL) using pretrain weights. TL details are reported in Online Supplement and Figure S3.^52^, ^53^ The models were trained utilizing NVIDIA GeForce‐RTX‐3060 6GB GPUs and torch python library.

### Performance metrics

To assess the performance of each model, accuracy, recall, precision, and F1 scores were calculated both overall and for each class. Accuracy is the ratio of correctly predicted true/false classifications among all results, and F1 score is a harmonic mean value that considers both precision and recall. Given the imbalance in class distribution, particularly in Step‐1, a weighted average F1 score was calculated. Additionally, receiver operating characteristic (ROC) and precision‐recall (PR) curves were plotted for each class using the models’ scores and a one‑vs‑all approach. Micro and weighted average curves represent overall model performance. Subsequently, the area under the curve (AUC) and the area under the precision‐recall curve (AUPRC) were calculated. Finally, the average values of the patients’ clinical characteristics were determined. Voluntary participation was ensured and informed written consent from all participants were obtained. This study was approved by the *Institutional Review Board of the University of Trieste* (approval code:70268).

## RESULTS

In our study 152 subjects were enrolled. Their characteristics are shown in Table 4. The original dataset included 1,196 AA (391 active, 237 inactive, and 568 regrowing), 300 FD, 606 FFA and LPP, 67 AGA, and 1,405 HV images. The test dataset contained 641 images and 240 images for Step‐1 and Step‐2, respectively. After the augmentation process, the number of images increased to a total of 49,219 for Step‐1, which included all disease pathologies. This comprised 39,368 images for the training set and 9,851 images for the test set. For the Step‐2, which involved different activity levels of AA, the dataset contained 23,885 images, with 19,076 images for the training set and 4,809 images for the test set. Tables 5 and 6 summarize the results of the trained models for Step‐1 and Step‐2, respectively. The performance measures were calculated on the test dataset excluding the augmented images. F1 score and accuracy are presented.

**TABLE 4:** Patient characteristics.

Variable		Results
Total number of patients n (%)		152 (100)
Gender	Female n (%)	105 (69.1)
	Male n (%)	47 (30.9)
Scalp disease	AA n (%)	58 (38.2)
	AGA n (%)	20 (13.2)
	FD n (%)	17 (11.1)
	LPP n (%)	19 (12.5)
Healthy Volunteers	HV n (%)	38 (25.0)

*Abbr*.: AA, alopecia areata; AGA, androgenetic alopecia; HV, healthy volunteers; FD, folliculitis decalvans; FFA, fibrosing frontal alopecia; LPP, lichen planopilaris; n, number

**TABLE 5:** Scores of network models for alopecia areata diagnostic classification – Step‐1.

Model 1 Disease	Metrics	DenseNet169	EfficientNetB0	ResNet152
Overall	Accuracy	87.36	88.92	86.27
Overall	F1 weighted	86.82	88.17	85.4
AA	Precision	89.87	89.88	87.92
	Recall	88.38	92.12	87.55
	F1	89.12	90.98	87.73
FFA‐LPP	Precision	86.55	90.09	89.38
	Recall	85.12	82.64	83.47
	F1	85.83	86.21	86.32
FD	Precision	91.66	94.83	91.53
	Recall	91.66	91.67	90
	F1	91.66	93.22	90.76
HV	Precision	84.23	86.1	82.02
	Recall	91.12	93.66	91.22
	F1	87.59	89.71	86.37
AGA	Precision	66.67	50	0
	Recall	14.29	7.14	0
	F1	23.53	12.5	0

*Abbr*.: AA, alopecia areata; AGA, androgenetic alopecia; HV, healthy volunteers; FD, folliculitis decalvans; FFA, fibrosing frontal alopecia; LPP, lichen planopilaris

**TABLE 6:** Scores of network models for alopecia areata activity level classification – Step‐2.

Model 2 Activity	Metrics	DenseNet169	EfficientNetB0	ResNet152
Overall	Accuracy	83.33	80.83	79.16
Overall	F1 weighted	83.36	80.75	79.13
AA active	Precision	79.27	90.47	76.00
	Recall	83.33	73.08	73.08
	F1	81.25	80.85	74.51
AA inactive	Precision	90.48	83.33	84.44
	Recall	79.16	72.92	79.17
	F1	84.44	77.78	81.72
AA in regrowth	Precision	83.72	75.56	79.16
	Recall	85.09	89.47	83.33
	F1	84.35	81.92	81.20

*Abbr*.: AA, alopecia areata

**FIGURE 3:**
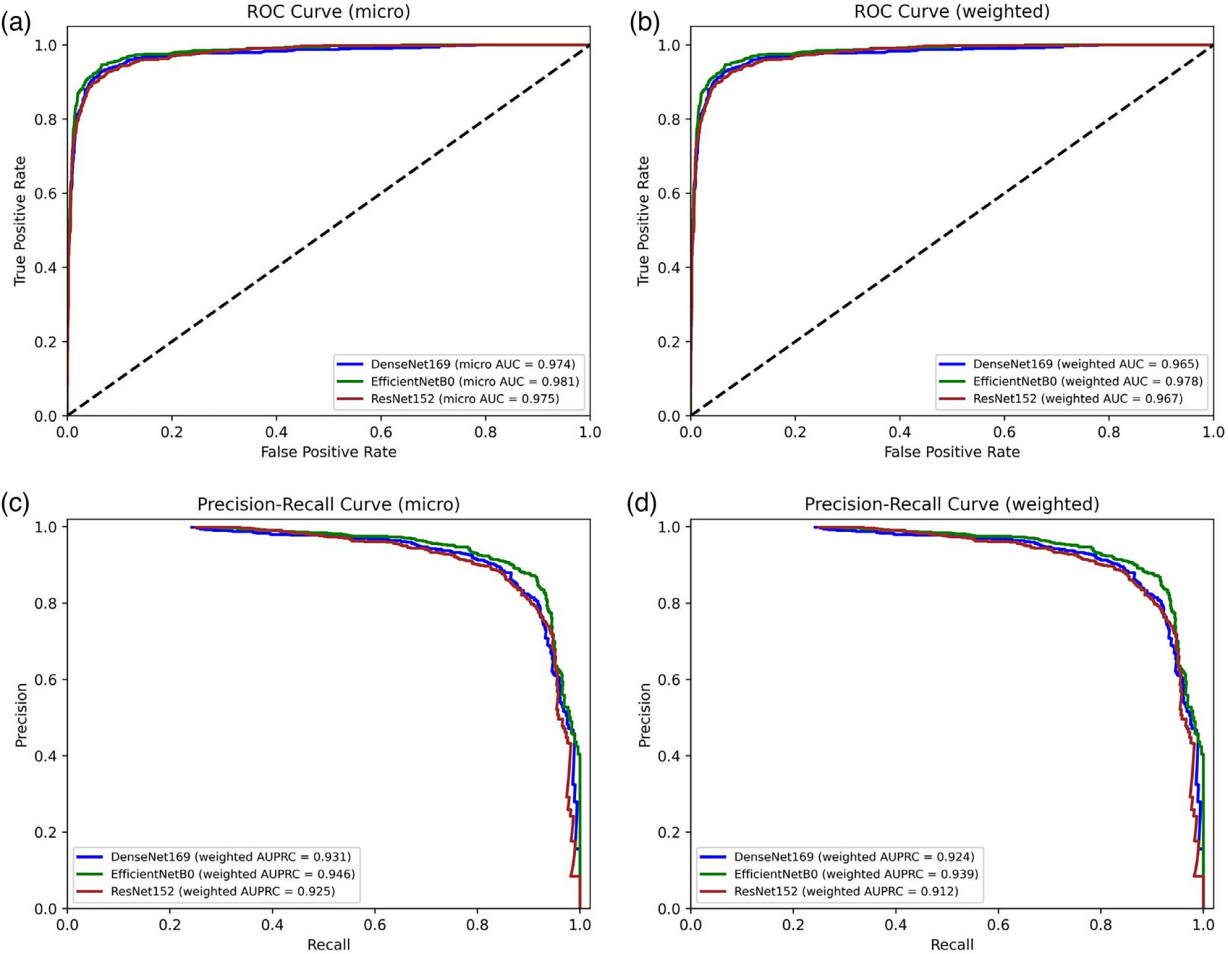
(a) Step 1 alopecia areata diagnostic classification receiver operating characteristic (ROC) curve (micro); (b) Step‐1 receiver operating characteristic (ROC) curve (weighted); (c) Step‐1 precision–recall curve (micro); (d) Step‐1 precision–recall curve (weighted).

**FIGURE 4:**
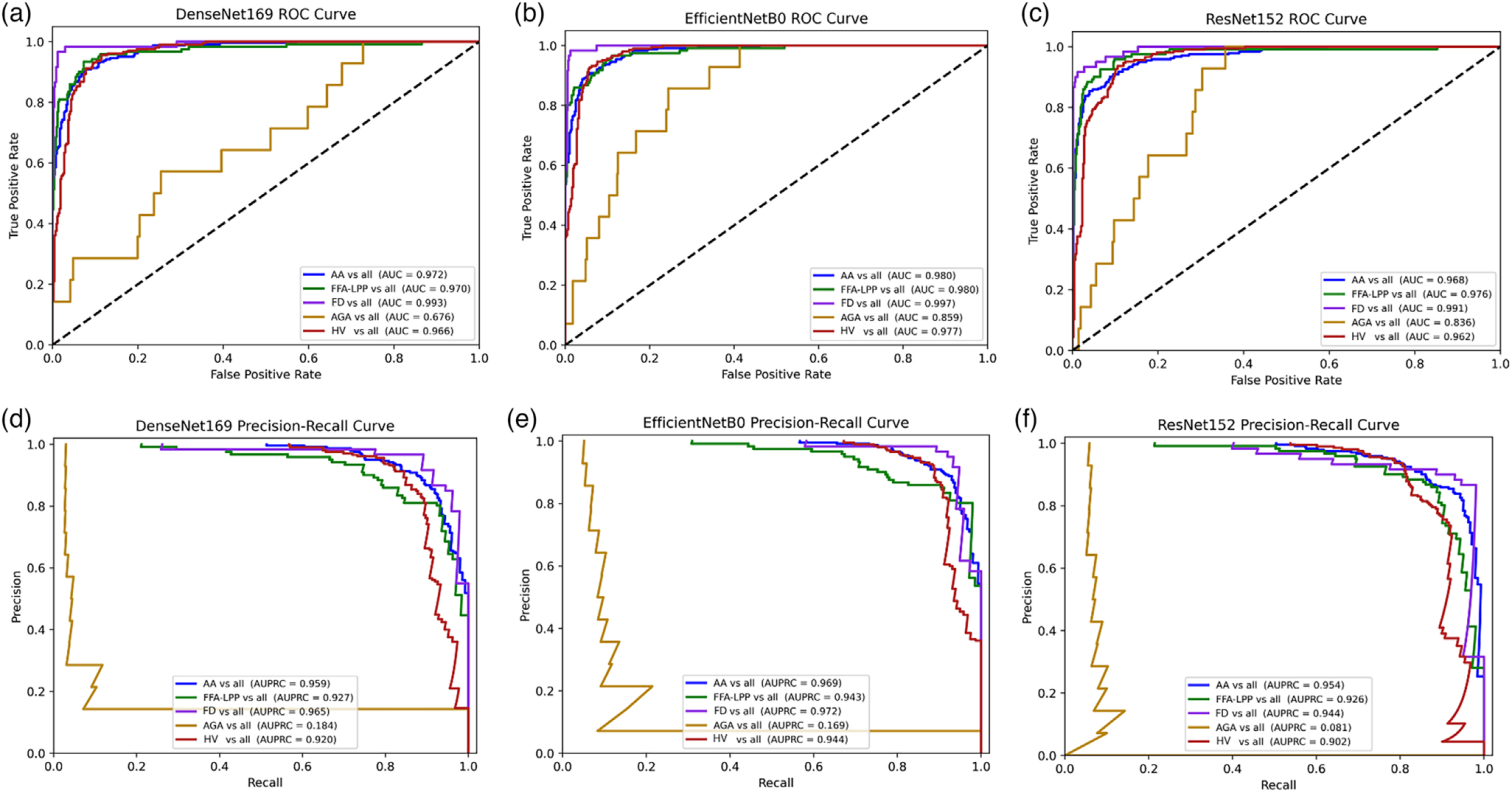
(a) Step‐1 alopecia areata diagnostic classification DenseNet169 model receiver operating characteristic (ROC) curve: each class vs all; (b) Step‐1 EfficientNetB0 receiver operating characteristic (ROC) curve: each class vs all; (c) Step‐1 ResNet152 receiver operating characteristic (ROC) curve: each class vs all; (d) DenseNet169 model precision–recall (PR) curve: each class vs all; (e) Step‐1 EfficientNetB0 precision–recall (PR) curve: each class vs all; (f) Step‐1 ResNet152 precision–recall (PR) curve: each class vs all.

In Step‐1, the highest overall results were demonstrated by the EfficientNetB0 model, with an overall accuracy of 88.92% and a weighted F1 score of 88.17%. The highest discriminatory capacity for AA disease was also obtained by this model, with F1 score of 90.98%, recall of 92.12%, and precision of 89.88%. Similar results were achieved for the FD, FFA‐LPP, and HV groups (Table 5). All models reached AUCs and AUPRCs > 0.90 (Figure 3), while the AUCs and AUPRCs for AA vs all were > 0.95 (Figure 4). However, significant differences in diagnostic capability were observed between conditions, particularly with AGA compared to others. The suboptimal performance in the AGA class can be attributed to its very low representation in the dataset (1.8% of the total images).

**FIGURE 5:**
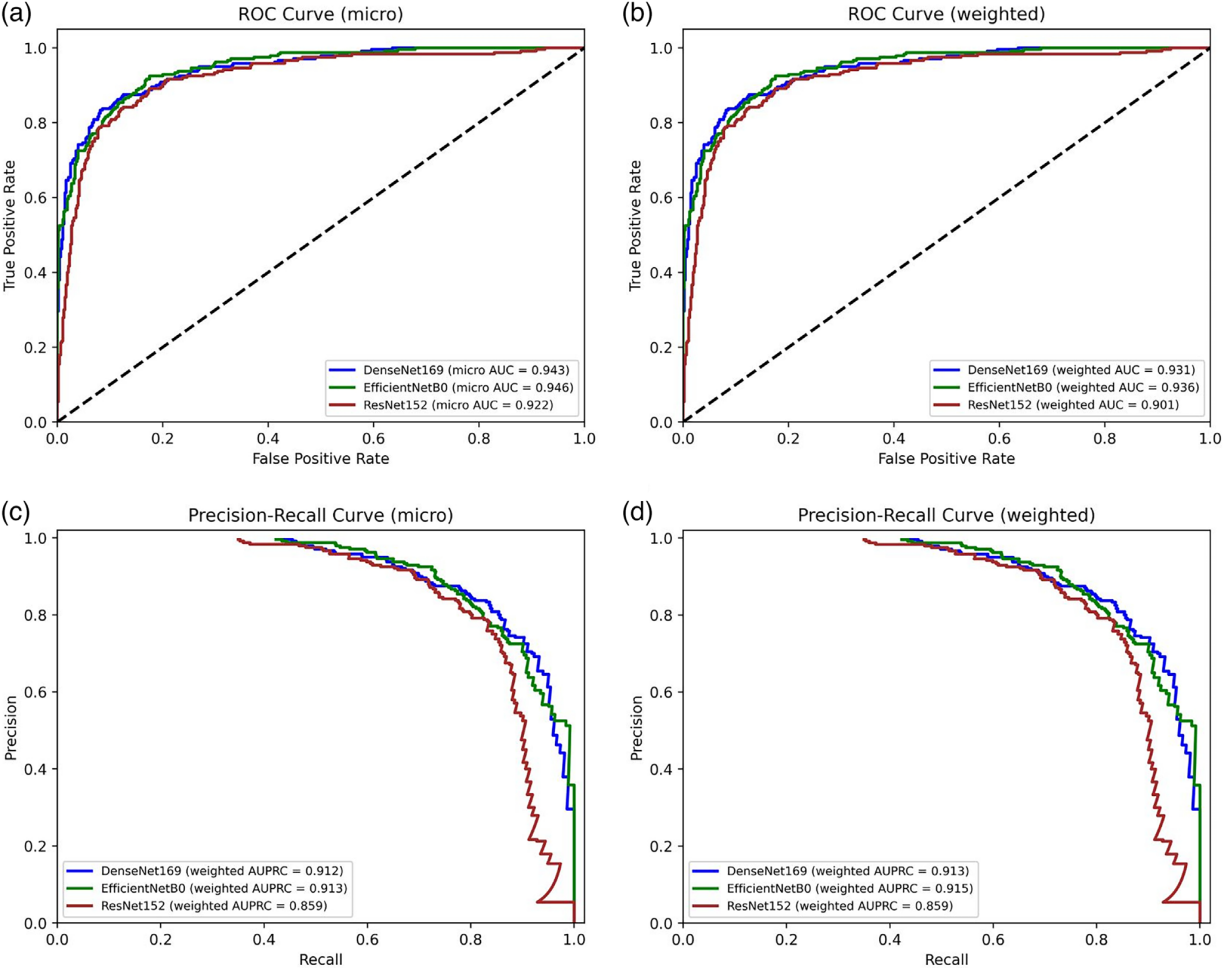
(a) Step‐2 alopecia areata activity level classification receiver operating characteristic (ROC) curve (micro); (b) Step‐2 receiver operating characteristic (ROC) curve (weighted); (c) Step‐2 precision–recall curve (micro); (d) Step‐2 precision–recall curve (weighted).

**FIGURE 6:**
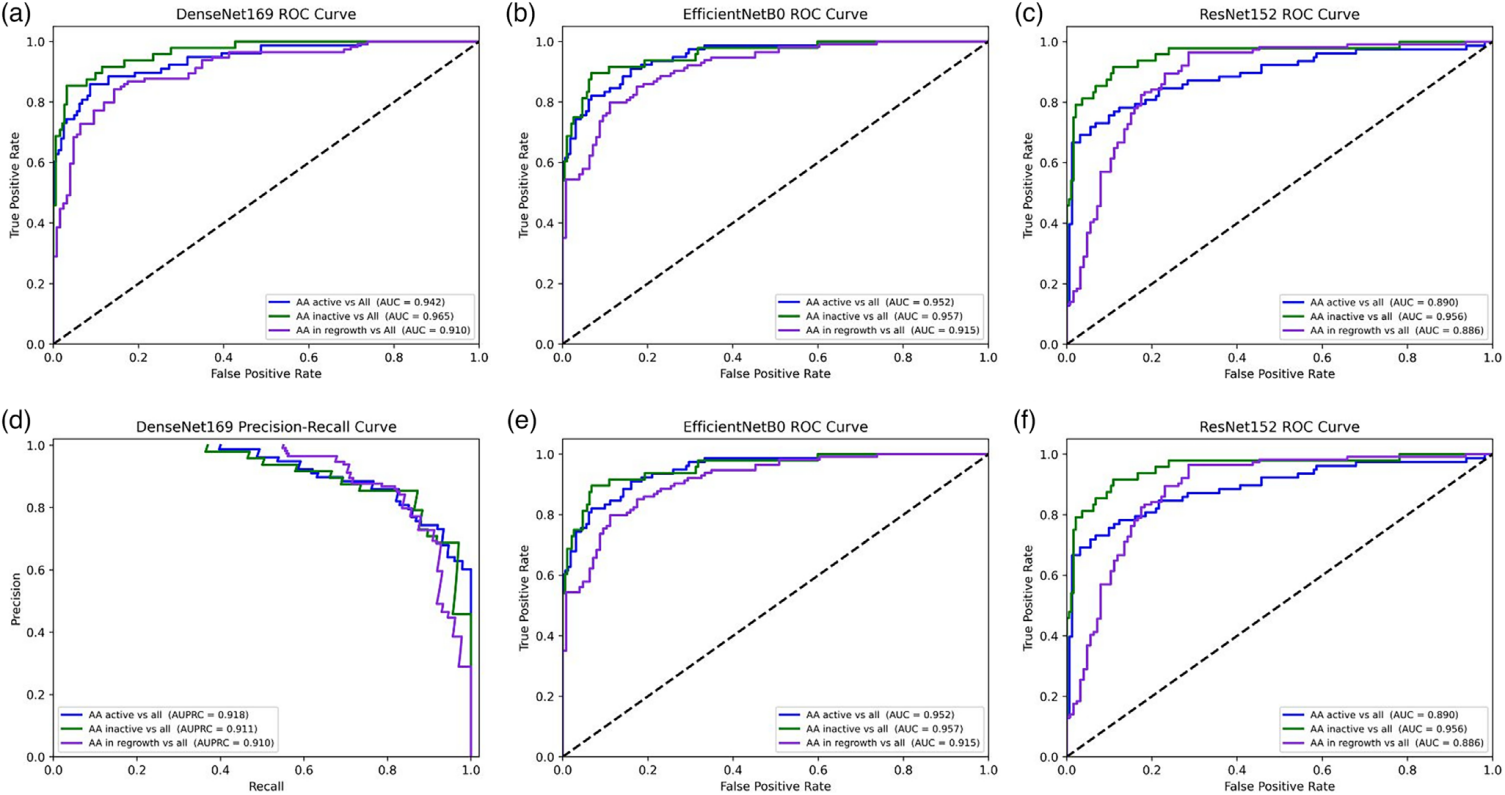
(a) Step‐2 alopecia areata activity level classification DenseNet169 model receiver operating characteristic (ROC) curve: each class vs all; (b) Step‐2 EfficientNetB0 receiver operating characteristic (ROC) curve: each class vs all; (c) Step‐2 ResNet152 receiver operating characteristic (ROC) curve: each class vs all; (d) DenseNet169 model precision–recall (PR) curve: each class vs all; (e) Step‐2 EfficientNetB0 precision–recall (PR) curve: each class vs all; (f) Step‐2 ResNet152 precision–recall (PR) curve: each class vs all.

In Step‐2, the best performance was observed with the DenseNet169 model, compared to EfficientNetB0 and ResNet152, resulting in an overall accuracy of 83.33% and a weighted F1 score of 83.36%. The model maintained balanced performance across each of the three groups: active AA with F1 score of 81.25%, inactive AA with F1 score of 84.44%, and in regrowth AA group with F1 score of 84.35% (Table 6). In Step‐2 DenseNet169 and EfficientNetB0 showed similar results, with AUCs and AUPRCs > 0.90, ResNet152 displayed AUC and AUPRC > 0.85 (Figure 5). Finally, in the activity analysis EfficientNetB0 and DenseNet169 models achieved higher AUC and AUPRC per each activity stage than ResNet152 (Figure 6).

## DISCUSSION

AI encompasses any technology enabling computers to replicate or surpass human decision‐making capabilities, tackling complex tasks independently or with minimal human involvement.^54^, ^55^, ^56^ In general terms, ML and DL, as its subsets, refer to the improvement of a computer‐program's performance through experience in relation to specific tasks and performance metrics.^9^, ^57^, ^58^ Artificial neural networks (ANN) are a specific type of ML model inspired by the structure and function of the human brain.^9^, ^57^, ^58^ Deep neural networks (DNN) typically consist of multiple hidden layers, organized in deeply nested network architectures. These characteristics make DNN particularly useful in domains with large and high‐dimensional data. To date, DL applications have been limited by the lack of large, labeled dermatologic image datasets.^9^, ^57^, ^58^ Although AI‐based classification systems cannot replace human experts, they can assist experts and enhance their ability to make accurate skin diagnoses and manage conditions by more than 33%, as reported in the literature.^59^ In fact, DL tools have the potential to improve workflow efficiency in several ways. They can quickly and efficiently provide AI‐assisted diagnoses and differential diagnoses, along with their associated probabilities, to assist primary care clinicians during patient evaluation. Additionally, these tools can relieve dermatologists from time‐consuming or repetitive tasks, such as detailed clinical assessments with dermoscopy. By automating these tasks, dermatologists can more effectively integrate and apply clinical data for higher‐order medical decision‐making, including the management of hair diseases.^57^

Among hair diseases, the prevalence of AA continues to rise annually, with its diagnosis primarily relying on clinical assessment supported by trichoscopy.^60^, ^61^ This tool can improve AA diagnosis by facilitating the evaluation of inflammatory activity, identification of severity indicators, assessment of therapeutic efficacy, and determination of prognostic factors.^34^ However, trichoscopy can be challenging due to the numerous conditions that may affect hair and the scalp, many of which share similar trichoscopic features (e.g., yellow dots), making diagnosis complex.^62^ Therefore, diagnosis is not based on a single trichoscopic feature but rather on a constellation of characteristics, complicating the recognition and differentiation of these diseases.^8^ These characteristics can change over time, depending on disease activity, stage, and response to medical treatment, further complicating the assessment of conditions that fluctuate or progress.^7^, ^34^, ^63^ Furthermore, patients may present with overlapping conditions or atypical clinical presentations.^64^, ^65^ As a result, proficiency with trichoscopy requires specialized training, presenting a hurdle for many practitioners.^8^ Consequently, the development of an AI‐model holds promise to assist dermatologists in diagnosing and staging AA and guiding medical management. In 2021, a framework was developed to distinguish between healthy hair and AA through clinical image classification.^66^ AI has also been explored in the severity assessment of AA through a DL framework. Indeed, Lee et al. demonstrated that dermatologists achieved significantly improved staging accuracy and interrater reliability using a computer‐assisted approach to determine the SALT score.^26^ This approach could enhance the explanatory power of the SALT score for predicting hair regrowth, making it more reliable for diverse clinical and research purposes.^67^ These findings suggest the importance of such models in guiding clinical management for different AA patients. Therefore, the development of a framework using videodermoscopy images may represent a helpful tool for differentiating AA from other diseases and recognizing markers of activity or inactivity. An AI model could assist in medical management by identifying signs of AA remission, which could help clinicians reduce or withdraw treatments or adopt a watch‐and‐wait approach. Conversely, in the presence of activity markers, AI could support the decision to maintain therapy or escalate from topical to systemic treatments.^68^ Another potential application of DL tools is in tele‐dermatology, which aims to overcome barriers to accessing specialist medical care. AI‐algorithms could allow peripheral centers to benefit from expert knowledge in specialized centers or could also enable self‐monitoring apps for tracking hair conditions.^59^, ^69^, ^70^, ^71^

Our study presents a two‐step algorithm to improve diagnosis using a set of data augmentation techniques suitable for scalp images and a TL approach. In Step‐1, we aimed to develop a model that distinguishes images of AA from other diseases, achieving overall accuracy of 88.92% and weighted F1 score of 88.17% using EfficientNetB0. Specifically, for the AA class, EfficientNetB0 achieved the highest F1 score of 90.98%. The model's performance was further demonstrated by ROC and PR curves. In Step‐2, the DenseNet169‐trained model showed the highest F1 score (83.36%) and accuracy (83.33%), with consistent results across all levels of disease activity. AUC and AUPRC were comparable between DenseNet169 and EfficientNetB0. Finally, the differences in diagnostic capability between AA and AGA compared to other conditions could be attributed to the limited number of AGA patients and, consequently, AGA images. Future work should focus on increasing the number of images for underrepresented conditions like AGA in our study.

To our knowledge, our model differs from those previously described in the literature by using videodermoscopic, rather than clinical, images, both to differentiate AA from healthy individuals and other trichological diseases and to assess AA at different stages of activity.^10^, ^11^, ^12^, ^13^, ^14^, ^15^, ^16^, ^17^, ^18^, ^19^, ^20^, ^21^, ^22^, ^23^, ^24^, ^25^, ^26^, ^27^, ^28^, ^29^, ^30^, ^31^ Limitations of this study include: *(1)* differences in diagnostic capacity among different scalp conditions due to an imbalanced dataset (e.g., limited AGA patients); *(2)* dataset gaps, such as the absence of diseases like tinea capitis or other cicatricial alopecias due to patient age inclusion criteria or a limited number of cases (e.g., LED); *(3)* limited generalizability across different prototypes due to the ethnic origin of our patient population; and *(4)* reliance on high‐end equipment (e.g., Medicam 1000^®^), which is not available in all hospitals, limiting broader telemedicine applications.

In conclusion, DL has immense potential in dermatology as an assistive diagnostic tool for skin diseases, with promising value in aiding diagnosis and disease quantification. This potential has already been demonstrated by dermatologist‐level accuracy in classifying numerous other skin lesions. To our knowledge, our study is the first to highlight the potential use of AI in trichoscopy for diagnosing and determining the activity level of AA. This application may enable more accurate diagnoses, improve patient care, and enhance dermatologists’ workflow. Further studies are needed to refine and validate these algorithms to improve patient care and safety, enhance dermatologist productivity, and expand access to high‐quality dermatologic care.

## CONFLICT OF INTEREST STATEMENT

All other authors declare no conflict of interest. R.D.C.C. has received honoraria for participation in speaker bureaus from Novartis, Eli Lilly, Sanofi and Pfizer. I.Z. is a member of a data safety monitoring board for Philogen and has received payment or honoraria for lectures, presentations, speaker bureaus, manuscript writing or educational events from Sanofi Genzyme, Sunpharma, Novartis, MSD, BMS, Philogen, Biogena, La Roche Posay, Kyowara Kirin, Fotofinder, Mallinckrodt, Cieffe Derma, Pierre Fabre, Regeneron, Canova, Almirall and Beiersdorf, as well as support for attending meetings and/or travel from Difa Cooper.

## Supporting information



Supplementry information

Supplementry information

Supplementry information

Supplementry information
