# Supplementary figures and images for: Analysis of trichoscopic images using deep neural networks for the diagnosis and activity assessment of alopecia areata – a retrospective study
Source: J Dtsch Dermatol Ges. 2025 Sep 30;24(1):44–55. doi: 10.1111/ddg.15847 (PMC12800882; doi:10.1111/ddg.15847)

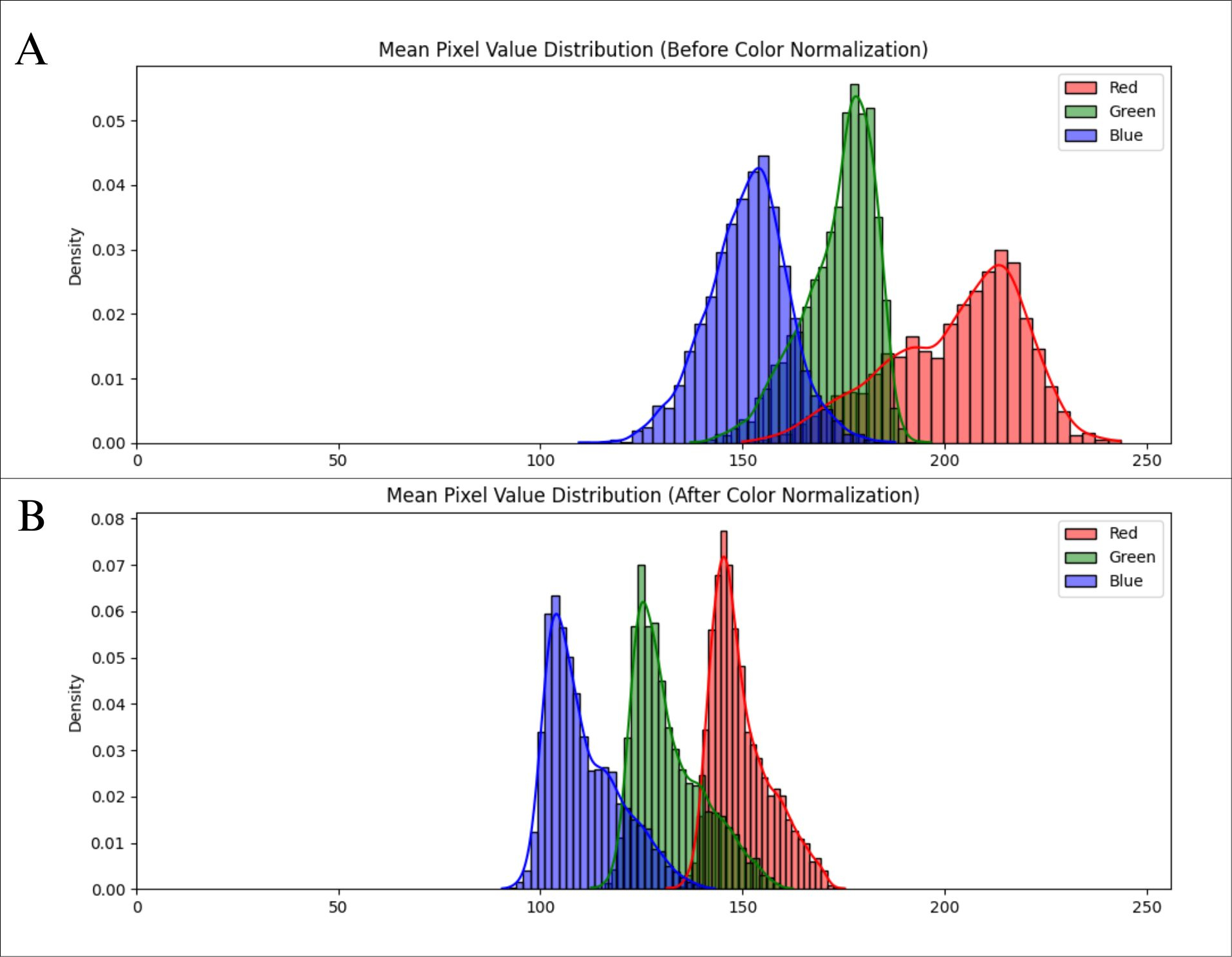

Supplement: Supplementary file 1 — Supplementry information [file DDG-24-44-s004.jpg]

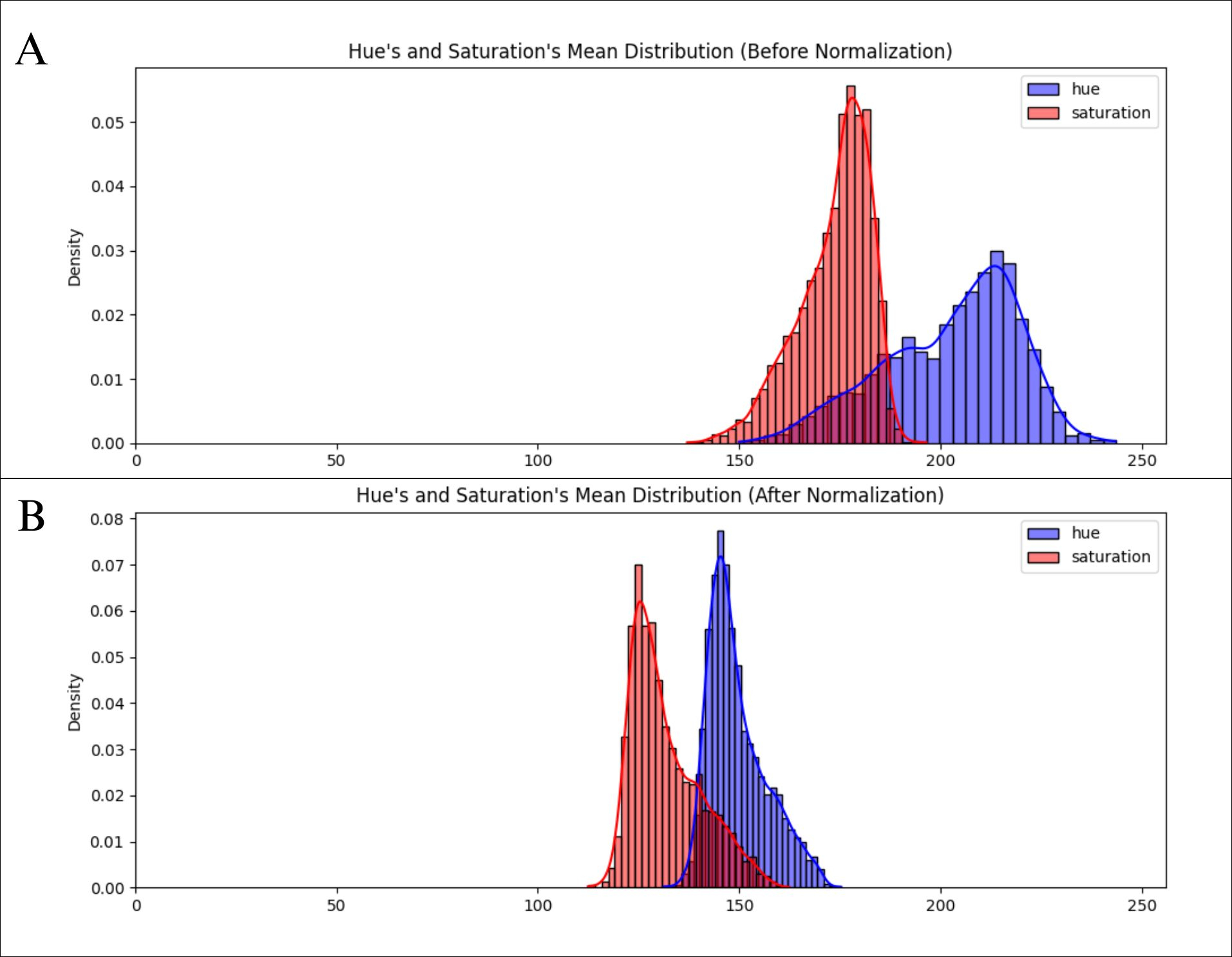

Supplement: Supplementary file 2 — Supplementry information [file DDG-24-44-s001.jpg]

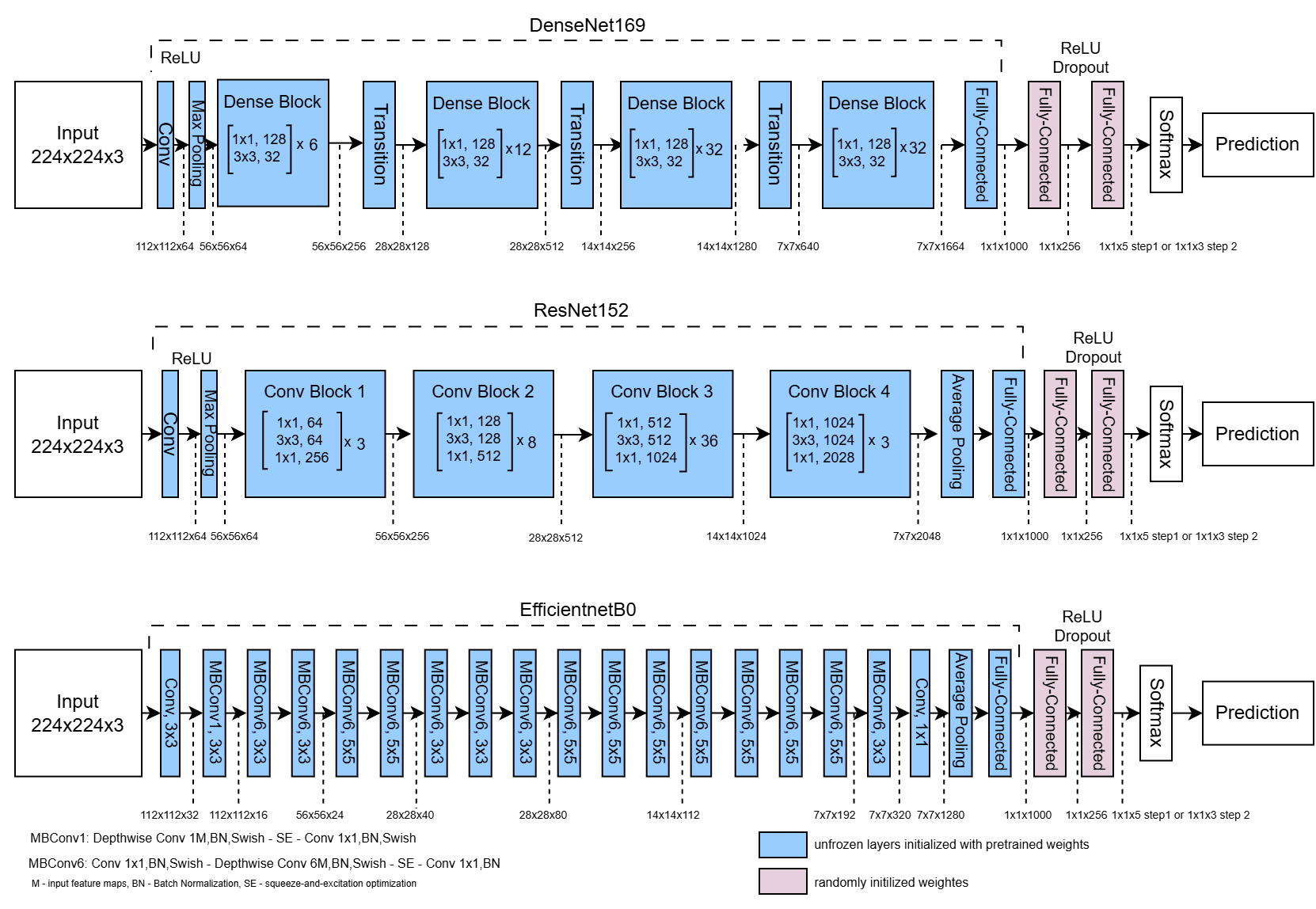

Supplement: Supplementary file 3 — Supplementry information [file DDG-24-44-s003.jpg]
