# Supplementary material for: Analysis of trichoscopic images using deep neural networks for the diagnosis and activity assessment of alopecia areata – a retrospective study
Source: J Dtsch Dermatol Ges. 2025 Sep 30;24(1):44–55. doi: 10.1111/ddg.15847 (PMC12800882; doi:10.1111/ddg.15847)
Supplement: Supplementary file 4 — Supplementry information [file DDG-24-44-s002.docx]

**Table S1.** Literature review of previous studies of AI-based diagnosis of AA assessment

| **Author** | **Year** | **Title** | **Objectives** | **Algorithm** | **Sample** |
| --- | --- | --- | --- | --- | --- |
| Shakeel CS, Khan SJ, Chaudhry B, at el ^12^ | 2020 | Classification framework for healthy hairs and alopecia areata (AA): a Machine Learning (ML) approach | To propose a framework pertaining to the classification of healthy hairs and AA based on hair images. | Support Vector machine (SVM) and k-nearest neighbor (KNN) classification model based on color, texture shape extracted features | 268 clinical hair images |
| Sayyad S, Midhunchakkaravarthy D, Sayyad F^13^ | 2022 | An Analysis of AA Classification Framework for Human Hair Loss Based on VGG-SVM Approach | To evaluate the efficacy of neural networks in recognizing alopecia and healthy hair | The VGG-19 pre-trained CNN model to extract features and SVM for classification task | 268 clinical scalp and hair images |
| Mittal A, Biswas DB, Karthikeyan U.^14^ | 2023 | Prediction of Alopecia Areata using CNN | To present a novel CNN architecture that can  facilitate and streamline the arena of detection Alopecia and healthy conditions | CNN architecture model | 400 scalp images |
| Saraswathi C, Pushpa B.^15^ | 2023 | FRCNN based Deep Learning for Identification and Classification of Alopecia Areata | To develop DL system to identify mild, moderate and severe AA and healthy hair | Faster Residual Convolutional Neural Network | 1158 clinical  hair and scalp images |
| Saraswathi C, Pushpa B.^16^ | 2023 | Machine Learning Algorithm for Classification of Alopecia Areata from Human Scalp Hair Images, in Computational Vision and Bio-Inspired Computing | To propose ML based classification of AA severity types from the human scalp hair images | An artificial algorithm for selecting features, then Modified extreme learning machine and Wavelet neural network were applied | 2058 clinical hair and scalp images |
| Saraswathi C, Pushpa B.^17^ | 2023 | AB-MTE Deep Classifier Trained with AAGAN for the Identification and Classification of Alopecia Areata | To propose DL based classification of AA severity types and healthy hair from the human scalp hair images | Attention-based Balanced Multitasking Ensembling Deep network in combination with AA-Generative Adversarial Network to augment data | 1158 clinical  hair and scalp images |
| Saraswathi C, Pushpa B.^18^ | 2023 | Multi-class support vector machine classification for detecting alopecia areata and scalp diseases. | To develop a method for image processing that incorporates a multiclass SVM classification approach of mild, moderate, severe AA and healthy hair | Multi-class SVM | 1158 clinical  hair and scalp images |
| Saraswathi C, Pushpa B.^19^ | 2023 | Attention balanced multi-dimension multi-task deep learning for alopecia recognition | To increase the accuracy of AA classification by learning local and global features across mild, moderate, severe AA and healthy hair. | An Attention-based Balanced Multi-Task Deep learning system | 1400 clinical  hair and scalp images |
| Saraswathi C, Pushpa B.^20^ | 2023 | Ensemble of pre-learned deep learning model and an optimized LSTM for Alopecia Areata classification | To propose for AA severity classification from both healthy human scalp hair and AA scalp hair images | Ensemble Pre-Learned DL and an Optimized Long Short-Term Memory | 1400 clinical  hair and scalp images |
| Kapoor I, Mishra A.^21^ | 2018 | Automated Classification Method for Early Diagnosis of Alopecia Using Machine Learning | To evaluate neural network for early prediction of AA and healthy patients based on collected features: length, nail brittleness, damage, hair follicle | The Feedforward ANN | Table data |
| Seo S, Park J.^22^ | 2020 | Trichoscopy of Alopecia Areata: Hair Loss Feature Extraction and Computation Using Grid Line Selection and Eigenvalue | To develop algorithm for extracting hair loss feature for scalp self-diagnosis | Grid line selection and eigenvalues analysis | Trichoscopic images |
| Ibrahim S., Noor Azmy Z. A., Abu Mangshor N. N. et el^23^ | 2020 | Pre-trained classification of scalp conditions using image processing | A study of pre-trained classification of scalp conditions (AA, dandruff, and normal scalp) using image processing techniques | Extracted features sorted in a Region of Interest (ROI) table and then SVM applied | 120 clinical scalp images |
| Roy M, Protity AT.^24^ | 2023 | Hair and scalp disease detection using machine learning and image processing. | To develop a DLapproach that predicts three main types of hair loss and scalp-related diseases: alopecia, psoriasis, and folliculitis. | 2D convolutional neural network model | 268 clinical scalp and hair images |
| Kim M, Gil Y, Kim Y, Kim J ^25^ | 2023 | Deep-Learning-Based Scalp Image Analysis Using Limited Data | To develop an approach for generating a model specialized for mild, moderate, severe AA and healthy hair analysis that achieves high accuracy by applying data preprocessing, data augmentation, and an ensemble of DLmodels | CNN models: ResNext101 DenseNet169, and XceptionNet41 | 18249  tricoscopic images |
| Lee S, Lee j, Choe S, et al^26^ | 2020 | Clinically applicable deep learning framework for measurement of the extent of hair loss in patients with Alopecia Areata | To develop a DL framework to determine the Severity of Alopecia Tool (SALT) score | AloNet that is based on U-Net segmentation DL network | 2716 images (18 patients) |
| Bernardis E, Castelo-Soccio L.^27^ | 2018 | Quantifying Alopecia Areata via texture analysis to automate the SALT score computation | To recreate the SALT scoring system in an automated way | Texture analysis was used to distinguish between normal hair and bald scalp. | 250 clinical images |
| Gudobba C, Mane T, Bayramova A, et al.^28^ | 2023 | Automating hair loss labels for universally scoring Alopecia from images: rethinking Alopecia scores. | To create an algorithmic quantification system (SALT score) for all hair loss, computational imaging analysis and algorithm design using retrospective image data | HairComb algorithm, which incorporates two encoder-decoder branches based on U-Net and ResNet50 | 250 participants, clinical images |
| Seol JE, Hong SM, Ahn SW, Jang SH, Kim H.^29^ | 2023 | Two-dimensional planimetry for Alopecia Areata severity evaluation compared with severity of alopecia tool: a pilot study. | To validate the SALT score through planimetric surface area measurement of alopecic patches | Image J program | 93 participants |
| Gao M, Wang Y, Xu H, et al ^30^ | 2022 | Deep Learning-based Trichoscopic Image Analysis and Quantitative Model for Predicting Basic and Specific Classification in Male Androgenetic Alopecia. | To develop a DL framework for automatic trichoscopic image analysis and a quantitative model for predicting basic and specific classification in male androgenetic alopecia | 2 types of CNN: the detection net (D-Net) structure for detecting hair follicle openings and the regression net (R-Net) structure for predicting the number of hairs and the proportion of vellus hairs, intermediate hairs and terminal hairs | 2910 trichoscopic images |
| Kim M, Kang S, Lee BD^32^ | 2022 | Evaluation of Automated Measurement of Hair Density Using Deep Neural Networks | To analyze the accuracy of hair density measurement by applying DL technology for object detection | EfficientDet, YOLOv4, and DetectoRS | 4492 trichoscopic images |
| Hoffmann R^33^ | 2003 | TrichoScan: a novel tool for the analysis of hair growth in vivo | To analyze automatically the biologic parameters of hair growth, which are: hair density; hair diameter; hair growth rate; and anagen/telogen ratio. | TrichoScan | Trichoscopic images |

*Data Normalization*

The original images were cropped into 1080-pixel squares on the left and right to remove camera shadows and noise, enhancing clarity and focusing on the scalp. Then, the variability of image colors was analyzed. The histograms display mean values distribution of the dataset by Red-Green-Blue (RGB) (**Figure S1**) and Hue-Saturation channels (**Figure S2**). The red, hue and saturation distributions before normalization demonstrate a more spread-out pattern, indicating significant variations in pixel intensity and color distribution among the images (**Figure S1A-S2A).**

To mitigate variations on the level of the pixels, we adopted the normalization approach described by Kim et al.  who demonstrated that the normalization in combination with data augmentation significantly increases the model performance.^25^ The normalization process involved using a reference image to determine the color index differences. This reference image served as a baseline for adjusting the brightness and color information in the other images. **Figure 2** displays the outcomes of the normalization procedure. The histograms after normalization demonstrate a shift in pixel distributions to a more standardized scale, with hue values becoming more condensed and aligned (**Figure S1B-S2B**). The distributions demonstrate that the normalization minimized variations of mean values.

The two datasets were divided into training and test sets with proportions of 80% and 20%, respectively, ensuring that both subsets maintain the same class distribution as the original datasets. The training set was utilized for model development while test set was used to verify the model performance out of sample. It should be noted that the highest accuracy is achieved when image transformation is applied to both training and test datasets.^45^

*Data Augmentation*

Considering that trichoscopy images can be obtained from different angles and various directions, the images were randomly flipped vertically and horizontally and rotated 90°, 180° or 270°. To distort the colors of the images the Principal Component Analysis (PCA) transformation was applied as proposed by Krixhevsky et al..^46^ PCA is a technique that transforms RGB pixel values into uncorrelated principal components that capture the most significant variance. For data augmentation, images are modified by adding multiples of these principal components, scaled by their corresponding eigenvalues with random parameter.^47^

Finally, the images were resized to 224x224 pixels as input data that allowed us to benefit from the use of a transfer learning (TL) approach.

*Models’ description*

We utilized three architectures of Convolutional Neural Network (CNN) models: ResNet152, DenseNet169, and EfficientNetB0.

- ResNet152 is a seminal DL model in which the weight layers learn residual functions with reference to the layer inputs. ResNet152 allows to reduce the error rate and to improve the performance, even in deeper networks.^48^ The deep network is built by utilizing skip connections which mitigate the vanishing gradient problem. We used the modification with the highest number of layers.^48,49^
- DenseNet169 is a type of CNN architecture that connects each layer to every other layer in a feed-forward fashion. Instead of traditional CNNs, where each layer has connections only to the subsequent layer, DenseNet169 has direct connections from any layer to all subsequent layers. This means that the feature maps of all preceding layers are used as inputs for each layer, and their own feature maps are used as inputs into all subsequent layers.^50^ The DenseNet169 consist of various layers including convolutional, max pool, dense, and transition layers.^50^
- The EfficientNetB0 model, introduced by Tan et al., utilizes a simple and compound scaling method to enhance CNN models.^50^ This approach scales network dimensions—depth, width, and resolution—in a balanced manner, resulting in high parameter efficiency and improved speed. The main building block of EfficientNetB0 is the mobile inverted bottleneck, which is based on the concept of MobileNet.^51^

*Transfer learning*

To overcome the issue of limited data, we employed the theory of transfer learning, which increases computational efficiency, and leverages the weights pre-trained on extensive datasets further enhancing performance. Georgakopoulos et al. compared two CNN models, one with random weight initialization and the other with pretrained weights on images and demonstrated that the latter approach significantly improved classification results.^52^ The referenced models were originally pre-trained on the ImageNet dataset containing more than 14 million annotated natural images, divided into 1000 categories.^53^ The TL approach and the models’ architecture are demonstrated in **Figure S3**. Removing the top layer, we appended a fully connected layer with 256 features with a Rectified Linear Unit (ReLU) activation function for each model. This layer includes dropout regularization to prevent overfitting. The final fully connected layer maps the 256 features to the number of output classes. For the first step, we set up 5 classes, and for the second step, we applied to 3 classes. Then, all layers were unfrozen and initialized with pre-trained weights except newly added layers, and backpropagation was run to train the new parameters. The models were optimized using the Adam optimizer with a step LR scheduler, the cross-entropy loss function, a batch size of 32, and 30 epochs utilizing NVIDIA GeForce-RTX-3060-6GB GPUs. The experiment was performed in the Python language utilizing the torch library.  The model parameters are summarized in **Table S2**.

**Table S2.** Training model parameters

|  | **Model** | **Optimization** | **Batch Size** | **Learning Rate** | **Weight Decay** | **Step Learning Rate** | |
| --- | --- | --- | --- | --- | --- | --- | --- |
|  |  |  |  |  |  | **Step size** | **Gamma** |
| **Step-1** | **DenseNet169** | Adam | 32 | 0.001 | 0 | 2 | 0.7 |
|  | **ResNet152** | Adam | 32 | 0.001 | 0 | 2 | 0.7 |
|  | **EfficientnetB0** | Adam | 32 | 0.001 | 0 | 2 | 0.7 |
| **Step-2** | **DenseNet169** | Adam | 32 | 0.001 | 0.001 | 2 | 0.7 |
|  | **ResNet152** | Adam | 32 | 0.001 | 0 | 2 | 0.7 |
|  | **EfficientnetB0** | Adam | 32 | 0.001 | 0 | 2 | 0.7 |

Step-1: Alopecia Areata diagnostic classification; Step-2: Alopecia Areata level of activity classification
